# Immunodominance and viral ditness in Gag may contribute to differential viral control in HLA-B*7 supertype individuals acutely infected with HIV-1C

**DOI:** 10.1186/1742-4690-9-S2-P160

**Published:** 2012-09-13

**Authors:** K Gounder, M Leshwedi, J Wright, M van der Stok, F Chonco, N Padayachee, B Ndimande, M Mokgoro, M Jaggernath, P Goulder, BD Walker

**Affiliations:** 1 University of KwaZulu-Natal, Durban, South Africa; 2 University of Oxford, Oxford, UK; 3 Ragon Institute of Massachusetts General Hospital, MIT and Harvard, Boston, MA, USA

## Background

HLA-B*7 supertype alleles are common among people of African descent and are associated with viral control. In particular, HLA-B*81 has been previously associated with reduced viral fitness. We analyzed the immunodominance of CD8+ T cell responses targeted by the B*7 supertype alleles, viral evolution and fitness dynamics over 1yr in acutely infected patients.

## Methods

Six HLA-B*7 supertype participants [HLA-B*81 (n=2), HLA-B*4201 (n=3) and HLA-B*4202 (n=1)] identified with acute HIV-1 infection (antibody negative, vRNA positive) in KwaZulu-Natal, South Africa were studied. CD8+ T cell responses were measured by the IFN-γ ELIspot assay. Replication capacities of viruses encoding Gag-protease were measured. Full-length HIV-1 Gag clonal sequencing of plasma was performed at ~14 days post infection and 1yr later.

## Results

The average viral set point of the 4 HLA-B*42 individuals was higher than the 2 HLA-B*81, 4.89 vs 4.16 respectively. Approximately 28 days after viral infection, CD8+ T cell responses were directed to an average of 2/5 (range 2-4) HLA-B*42 Gag-specific epitopes, median magnitude of 490 (range 170–2,480 SFC/million PBMCs). None of these 4 individuals had selected for escape mutations in the immunodominant TL9 epitope at 1yr post-infection. Interestingly, CD8+ T cell responses were only against the TL9 epitope for the 2 HLA-B*81 patients with a median magnitude of 950 (range 300–1780 SFC/million PBMCs). One patient had a single wild type epitope in the transmitted virus, compared to 4/5 wild type epitopes in the second patient. However, CD8+ T cell responses were only elicited at the TL9 epitope with a low magnitude against T186S in the 1 patient with a much lower viral fitness.

## Conclusion

Strong, rather than broad immunodominant responses in HLA-B*7 individuals is desirable in viral control. Furthermore, this study emphasizes the advantage of early dominant CD8+ T cell immune responses and an attenuated virus in conferring clinical benefit among HLA-B*7 supertype individuals.

